# Berufsbedingte Unterschiede bei COVID-19-Morbidität und -Mortalität in Deutschland. Eine Analyse von Krankenkassendaten von 3,17 Mio. Versicherten

**DOI:** 10.1007/s00103-023-03738-9

**Published:** 2023-07-19

**Authors:** Morten Wahrendorf, Valerie Schaps, Marvin Reuter, Jens Hoebel, Benjamin Wachtler, Josephine Jacob, Marco Alibone, Nico Dragano

**Affiliations:** 1 Institut für Medizinische Soziologie, Centre for Health and Society, Medizinische Fakultät und Universitätsklinikum, Heinrich-Heine-Universität Düsseldorf, Düsseldorf, Deutschland; 2 Juniorprofessur für Soziologie, insb. Arbeit und Gesundheit, Fakultät für Wirtschafts- und Sozialwissenschaften, Otto-Friedrich-Universität Bamberg, Bamberg, Deutschland; 3 Fachgebiet Soziale Determinanten der Gesundheit, Abteilung für Epidemiologie und Gesundheitsmonitoring, Robert Koch-Institut, Berlin, Deutschland; 4 InGef – Institut für angewandte Gesundheitsforschung, Berlin GmbH, Berlin, Deutschland

**Keywords:** COVID-19, SARS-CoV-2, Berufliche Ungleichheiten, Sozialepidemiologie, Deutschland, COVID-19, SARS-CoV‑2, Occupational inequalities, Social epidemiology, Germany

## Abstract

**Einleitung:**

Dem Beruf wurde während der COVID-19-Pandemie eine zentrale Rolle beim Infektions- und Krankheitsgeschehen zugesprochen. Für Deutschland liegen jedoch bisher nur wenige Auswertungen zu berufsbedingten Unterschieden bei COVID-19-Erkrankungsrisiken, COVID-19-assoziierten Krankenhausaufenthalten und Mortalität vor.

**Methoden:**

Die Studie nutzt longitudinale Krankenkassendaten der Forschungsdatenbank des Instituts für angewandte Gesundheitsforschung (InGef) von 3,17 Mio. Versicherten zwischen 18 und 67 Jahren (1.488.452 Frauen, 1.684.705 Männer). Outcomes (Erkrankungsrisiko, Hospitalisierung und Mortalität) wurden durch übermittelte COVID-19-Diagnosen zwischen dem 01.01.2020 und 31.12.2021 bestimmt. Berufe wurden entlang von 4 Gruppierungen der amtlichen Systematik der Klassifikation der Berufe unterschieden. Neben kumulativen Inzidenzen bestimmen wir relative Risiken (RR) – jeweils getrennt für Männer und Frauen.

**Ergebnisse:**

Erkrankungsrisiken sind in personenbezogenen Dienstleistungsberufen erhöht, insbesondere in Gesundheitsberufen im Vergleich zu den übrigen Berufen (RR für Frauen 1,46; für Männer 1,30). Ähnliches gilt für soziale und kulturelle Dienstleistungsberufe (allerdings nur bei Frauen) und für Fertigungsberufe (nur bei Männern). Zudem sind die Risiken für Krankenhausaufenthalte und Mortalität in Reinigungsberufen sowie in Verkehrs- und Logistikberufen (v. a. für Männer) erhöht. Für alle 3 Outcomes sind die Risiken in Berufen ohne Leitungsfunktion und entlang des Anforderungsniveaus höher (am höchsten für Helfertätigkeiten und am niedrigsten für Expertentätigkeiten).

**Schlussfolgerung:**

Die Studie liefert wichtige Befunde zu berufsbedingten und geschlechtsabhängigen Unterschieden bei COVID-19-Morbidität und -Mortalität in Deutschland, die Ansatzpunkte für strukturelle Infektionsschutzmaßnahmen aufzeigen.

**Zusatzmaterial online:**

Zusätzliche Informationen sind in der Online-Version dieses Artikels (10.1007/s00103-023-03738-9) enthalten.

## Abstract (en)

**Introduction:**

During the COVID-19 pandemic, occupation was assumed to play a central role in the occurrence of infection and disease. For Germany, however, there are only a few studies that analyse occupational differences in risk of COVID-19, COVID-19-associated hospitalisation, and mortality.

**Methods:**

The study uses longitudinal health insurance data from the research database of the Institute for Applied Health Research (InGef) with information on 3.17 million insured persons aged 18–67 years (1,488,452 women; 1,684,705 men). Outcomes (morbidity, hospitalisation, and mortality) were determined on the basis of submitted COVID-19 diagnoses between 1 January 2020 and 31 December 2021. Occupations were classified according to four groupings of the official German classification of occupations. In addition to cumulative incidences, relative risks (RR) were calculated – separately for men and women.

**Results:**

There is an increased risk of disease in personal service occupations, especially in health care, compared to other occupations (RR for women 1.46; for men 1.30). The same applies to social and cultural service occupations (but only for women) and for manufacturing occupations (only for men). In addition, the risks for hospitalisation and mortality are increased for cleaning occupations and transport and logistics occupations (especially for men). For all three outcomes, the risks are higher in non-managerial occupations and differ by skill level (highest for unskilled jobs and lowest for expert positions).

**Conclusion:**

The study provides important findings on work- and gender-related differences in COVID-19 morbidity and mortality in Germany, which indicate starting points for structural infection protection measures.

## Einleitung

Auch 3 Jahre nach Beginn der COVID-19-Pandemie fehlen für Deutschland bevölkerungsweite Untersuchungen zur Frage, ob bestimmte Berufsgruppen besonders häufig von COVID-19 und schweren Erkrankungsverläufen betroffen waren. Dies ist umso erstaunlicher, da dem Beruf eine zentrale Rolle beim Infektions- und Krankheitsgeschehen zugesprochen wurde [1–3] und berufsspezifische Interventionen ein erhebliches Präventionspotenzial hinsichtlich der Eindämmung des Infektionsgeschehens und Verringerung der Krankheitslast für zukünftige Pandemien haben könnten. Durch den vorliegenden Beitrag soll die Forschungslage zu berufsbedingten Unterschieden bei COVID-19-Morbidität und -Mortalität in Deutschland verbessert werden. Grundlage ist eine Auswertung von Krankenkassendaten von über 3 Mio. gesetzlich Versicherten mit Informationen zu COVID-19-Erkrankungen und -Krankheitsverläufen (genauer: COVID-19-assoziierte Krankenhausaufenthalte und Mortalität).

Dass bestimmte Berufe sowohl ein erhöhtes Risiko für eine SARS-CoV-2-Infektion als auch für einen schweren COVID-19-Verlauf haben, wurde bereits zu Beginn der Pandemie schnell deutlich. Hierzu gibt es zahlreiche Übersichtsarbeiten [3–8]. Die ersten Studien stammten dabei aus den USA und England und konzentrierten sich vor allem auf sogenannte systemrelevante Berufe (englisch: „essential workers“). Dabei zeigten sich insbesondere höhere Infektionsrisiken bei Berufen im Gesundheitswesen (v. a. Pflege; [9]), aber auch in den Bereichen „Transport und Verkehr“ oder im produzierenden Gewerbe (v. a. Nahrungsmittelherstellung; [10]). Ähnliches zeigte sich auch in Untersuchungen zur Verlaufsschwere, in denen insbesondere während der ersten Pandemiephase (Frühjahr 2020) ein erhöhtes Risiko für Krankenhausaufenthalte oder Mortalität bei Personen in Gesundheitsberufen und bei SozialarbeiterInnen berichtet wurde [11]. Die meisten Studien konzentrierten sich allerdings auf relativ homogene Bevölkerungsgruppen (wie etwa bei seroepidemiologischen Studien in einzelnen Berufsgruppen) sowie auf lediglich einen kurzen Zeitraum der Pandemie. Damit liegt nur eine kleine Zahl von Studien vor und eine umfassende Untersuchung verschiedener Berufsgruppen für längere Zeiträume auf Basis breiter bevölkerungsbasierter Daten ist nicht möglich. Dies gilt insbesondere für Untersuchungen der Schwere eines COVID-19-Verlaufs (z. B. durch die Datenerhebung von Krankenhausaufenthalten), da sie in der Allgemeinbevölkerung insgesamt eher selten sind und deren Untersuchung größere Stichproben erfordert. Interessant ist eine Studie zum Infektionsgeschehen während der ersten (Februar bis Juli 2020) und zweiten Welle (Juli bis Dezember 2020) der Pandemie in Norwegen auf Basis von Registerdaten der Gesamtbevölkerung [12]. Diese zeigt ebenfalls, dass Personen in Gesundheitsberufen im Vergleich zur Allgemeinbevölkerung wesentlich höhere Infektionsrisiken haben, aber auch, dass dies vor allem in der ersten Welle der Fall war. Während der zweiten Welle waren hingegen vor allem Beschäftigte in der Gastronomie und LehrerInnen betroffen. Für Deutschland liegt unseres Wissens bisher nur eine einzige bevölkerungsbasierte Studie zum Infektionsrisiko vor, die auf Basis von Daten der NAKO-Gesundheitsstudie das (selbstberichtete) Infektionsrisiko während der ersten Pandemiewelle analysiert [13]. Die Ergebnisse zeigen höhere Infektionsrisiken bei personenbezogenen Dienstleistungsberufen, aber interessanterweise auch bei Berufen mit hohem Qualifikationsniveau oder für Führungskräfte. Letzteres deckt sich mit Beobachtungen anderer Studien, nämlich dass zu Beginn der Pandemie die Infektionsraten in sozial benachteiligten Regionen teilweise niedriger waren und sich erst im Verlauf zu Ungunsten dieser benachteiligten Regionen umkehren (siehe hierzu auch [4, 14]). Dies unterstreicht, dass sich die Anfangszeit der Pandemie hinsichtlich gefundener Zusammenhänge von den übrigen Pandemiephasen unterscheiden könnte – vielleicht aufgrund höherer Reisemobilität (in Berufen mit hohem Qualifikationsniveau oder für Führungskräfte) vor Pandemiebeginn.

Es liegen somit für Deutschland nur begrenzt Studien zu beruflichen Unterschieden bei COVID-19-Erkrankungen und zur Verlaufsschwere vor und es fehlen Studien, die beides auch über die erste Pandemiephase hinaus betrachten. Insgesamt fehlen bisher auch Betrachtungen, die Geschlechterunterschiede im Beruf berücksichtigen, wie z. B. den höheren Anteil von Frauen in der Pflege innerhalb der Gesundheitsberufe. Ziel des vorliegenden Beitrags es ist daher, berufliche Unterschiede hinsichtlich der Risiken einer COVID-19-Erkrankung, eines COVID-19-bedingten Krankenhausaufenthaltes sowie von COVID-19-assoziierter Mortalität für Männer und Frauen in Deutschland umfassend zu beschreiben. Als Datenbasis dienen Daten der gesetzlichen Krankenversicherung von mehr als 3,17 Mio. Versicherten über einen Zeitraum von 2 Jahren (2020 und 2021).

## Methoden

### Datenbasis

Die Daten stammen aus der Forschungsdatenbank (FDB) des Instituts für angewandte Gesundheitsforschung (InGef), welche bereits an anderer Stelle ausführlich beschrieben wurde [15]. Kurz zusammengefasst verfügt sie über anonymisierte Abrechnungsdaten im Längsschnitt (seit 2014) von rund 8,8 Mio. gesetzlich Versicherten, die von ca. 60 Krankenkassen (hauptsächlich Betriebs- oder Innungskrankenkassen) in ganz Deutschland an das InGef übermittelt werden. Dabei liegen neben Stammdaten der Versicherten (inkl. Geschlecht und Alter) auch detaillierte Daten zum Krankheitsverlauf (in Form von ICD-Codes), Informationen zu ambulanten und stationären Behandlungen sowie zu Arzneimittelverschreibungen und zur Arbeitsunfähigkeit vor. Dazu kommen vom Arbeitgeber übermittelte Details zum Beruf (auf Basis des Tätigkeitsschlüssels (TTS), s. unten).

### Datenschutz und Ethik

Die Daten liegen anonymisiert vor und ermöglichen keine Rückschlüsse auf einzelne Versicherte, einzelne Leistungserbringer (z. B. ÄrztInnen, Praxen, Krankenhäuser, Apotheken) oder auf den Bestand einzelner Krankenkassen. Die Auswertungen durch InGef erfolgen ausschließlich in geschützter Umgebung (eigenes sicheres Laufwerk) gemäß geltenden Datenschutzrichtlinien (Bundesdatenschutzgesetz) sowie Empfehlungen der Guten Epidemiologischen Praxis (GEP) und der Guten Praxis Sekundärdatenanalyse (GPS). Die anonymisierte Datenanalyse erfolgt mit Zustimmung der Ethikkommission an der Medizinischen Fakultät der Heinrich-Heine-Universität Düsseldorf (Studien-Nr.: 2021-1710) im Einklang mit nationalem Recht sowie gemäß der Deklaration von Helsinki von 1975 (in der aktuellen, überarbeiteten Fassung).

### Beobachtungszeitraum

Der Beobachtungszeitraum vom 01.01.2020 bis zum 31.12.2021 umfasst insgesamt 2 Jahre und somit die 4 Hauptinfektionswellen seit Pandemiebeginn – entsprechend der Phaseneinteilung des Robert Koch-Instituts [16, 17].

### Studienpopulation

Als Studienbasis dienen Daten der Versicherten, die am 01.01.2020 bei einer in der InGef-FDB enthaltenen Krankenkassen versichert waren (6,8 Mio.). Hiervon für die weitere Analyse eingeschlossen wurden nur Personen, die zu Beobachtungsbeginn im Erwerbsalter (18 bis 67 Jahre) sowie während des Beobachtungszeitraums durchgängig versichert waren bzw. in diesem Zeitraum verstorben sind. Ausgeschlossen wurden Versicherte, für die keine Berufsmerkmale auf Basis eines TTS ermittelt werden konnten. Daraus ergibt sich eine Nettostichprobe von 3.173.157 Versicherten (1.488.452 Frauen und 1.684.705 Männer). Dies entspricht ca. 10 % aller sozialversicherungspflichtig Beschäftigten in Deutschland im Januar 2020 (rund 33,6 Mio.; [18]). Einzelheiten zur Auswahl der Studienpopulation sind als Flussdiagramm im Onlinematerial (Abbildung S1) zusammengefasst.

### COVID-19-bezogene Outcomes

Untersucht wurden die 3 COVID-19-bezogenen Outcomes:

- *COVID-19-Erkrankungsrisiko: *Eine Erkrankung an COVID-19 war hierbei definiert durch mindestens eine gesicherte Diagnose im Beobachtungszeitraum anhand des ICD-Codes U07.1! (COVID-19 laborbestätigt) – entweder ambulant oder als Haupt- oder Nebendiagnose im Rahmen einer stationären Versorgung
- *COVID-19-bedingter Krankenhausaufenthalt*, definiert als eine vollstationäre Krankenhausbehandlung, in dem die Diagnose U07.1! bei Aufnahme oder als Haupt- oder Nebendiagnose im Beobachtungszeitraum dokumentiert wurde
- *COVID-19-assoziierte Mortalität: *Hierzu zählen alle Todesfälle im Beobachtungszeitraum, die entweder innerhalb von 30 Tagen nach einer nachgewiesenen COVID-19-Erkrankung auftraten (ambulante COVID-19-Erkrankung und -Behandlung), die während eines COVID-19-bedingten Krankenhausaufenthalts verstarben oder die innerhalb von 14 Tagen nach einem vollstationären Krankenhausaufenthalt mit Haupt- oder Nebendiagnose U07.1! auftraten (basierend auf dem Entlassungsdatum)

### Berufsmerkmale

Zur Bestimmung des Berufs nutzen wir 4 unterschiedliche Gruppierungen, die allesamt auf dem in der FDB zuletzt dokumentierten TTS beruhen (zwischen 2014 und 2019). Dieser TTS besteht aus insgesamt 9 Ziffern, die vom Arbeitgeber routinemäßig an die Krankenkassen übermittelt werden, wobei die ersten 5 Stellen gemäß der „Klassifikation der Berufe 2010“ (KldB 2010) kodiert sind und Details zur ausgeübten Tätigkeit enthalten [19, 20]. Beispiele sind der Code „81302“ (Gesundheits- und Krankenpfleger) oder „81393“ (Stationsleiter/Krankenpflege). Mit den ersten beiden Stellen der KldB können Berufssektoren (5 Kategorien) und Berufssegmente (14 Kategorien) bestimmt werden. Mit Hilfe der 4. und 5. Stelle lassen sich 2 Arten der Leitungsfunktion von Berufen ohne Leitungsfunktion abgrenzen (3 Kategorien) und die 5. Stelle enthält Informationen zum Anforderungsniveau (4 Kategorien). Berufssektoren und Berufssegmente ordnen Berufe in erster Linie nach berufsfachlichen Kriterien ein (d. h. horizontale Dimension mit berufsfachlicher Nähe innerhalb der Kategorien) und sind die 2 größtmöglichen Aggregationsstufen der KldB 2010. Anforderungsniveau und Leitungsfunktion beschreiben hingegen eine vertikale Struktur von Berufen entlang unterschiedlicher Komplexitätsgrade eines Berufes bzw. nach der Art der beruflichen Leitungsfunktion (Führungskräfte oder Aufsichtskräfte). Eine ausführliche Beschreibung der KldB 2010 und ihrer konzeptionellen Grundlagen findet sich an anderer Stelle [19, 20]. Die einzelnen Ausprägungen der Gruppierungen und deren Verteilung in der Studienpopulation zeigt Tab. 1.

**Table 1:** 

		Frauen (*n* = 1.488.452)		Männer (*n* = 1.684.705)	
–	Kategorie	Anzahl	%	Anzahl	%
Berufssektoren^a^	Produktionsberufe	120.429	8,4	779.769	47,1
	Personenbezogene Dienstleistungsberufe	470.102	32,6	141.547	8,5
	Kaufmännische und unternehmensbez. Dienstleistungsberufe	704.986	48,9	391.635	23,6
	IT- und naturwiss. Dienstleistungsberufe	31.543	2,2	100.682	6,1
	Sonstige wirtschaftliche Dienstleistungsberufe	113.678	7,9	243.414	14,7
Berufssegmente^a^	Land‑, Forst- und Gartenbauberufe	9455	0,7	19.051	1,1
	Fertigungsberufe	36.686	2,5	194.998	11,8
	Fertigungstechnische Berufe	64.301	4,5	436.764	26,4
	Bau- und Ausbauberufe	9987	0,7	128.956	7,8
	Lebensmittel- und Gastgewerbeberufe	68.596	4,8	45.202	2,7
	Med. u. nicht-med. Gesundheitsberufe	230.748	16,0	42.062	2,5
	Soziale und kulturelle Dienstleistungsberufe	170.758	11,9	54.283	3,3
	Handelsberufe	182.033	12,6	112.225	6,8
	Berufe in Unternehmensführung und -organisation	298.055	20,7	162.503	9,8
	Unternehmensbezogene Dienstleistungsberufe	224.898	15,6	116.907	7,1
	IT- und naturwiss. Dienstleistungsberufe	31.543	2,2	100.682	6,1
	Sicherheitsberufe	9264	0,6	24.789	1,5
	Verkehrs- und Logistikberufe	57.226	4,0	206.225	12,4
	Reinigungsberufe	47.188	3,3	12.400	0,7
Anforderungsniveau^a^	HelferInnen	199.552	13,9	181.903	11,0
	Fachkräfte	929.056	64,5	983.958	59,4
	SpezialistInnen	173.104	12,0	269.922	16,3
	ExpertInnen	139.066	9,7	221.272	13,4
Leitungsfunktion	Aufsichtskräfte	18.517	1,2	60.888	3,6
	Führungskräfte	21.548	1,4	55.557	3,3
	Keine Leitungsfunktion	1.448.387	97,3	1.568.260	93,1

^a^Für 2,4 % der Versicherten liegen keine verwertbaren Informationen zum Berufssektor oder Berufssegment vor (*n* = 75.372). Gleiches gilt für Anforderungsniveau (*n* = 75.324)

### Statistische Methoden

Alle Analysen erfolgten getrennt für Männer und Frauen, beginnend mit einer Beschreibung der Studienpopulation (Tab. 1) und einem Vergleich der Verteilung der Berufsmerkmale mit offiziellen Daten der Bundesagentur für Arbeit (Tabelle S1 im Onlinematerial). Als Nächstes wurden jeweils getrennt für die 3 Outcomes (Tab. 2, 3 und 4) Inzidenzen bzw. Mortalitätsraten entlang der 4 Berufsmerkmale betrachtet. Neben absoluten Zahlen (Anzahl Exponierter und Anzahl von Fällen) berechneten wir kumulative Inzidenzen (Fälle pro 100.000 Versicherte) über den Beobachtungszeitraum. Außerdem berechneten wir auf deren Basis relative Risiken (plus 95 %-Konfidenzintervalle). Anstatt eine bestimmte Berufsgruppe als Referenzgruppe bei der Bestimmung der relativen Risiken zu wählen (und damit die Risiken nur im Vergleich zu der gewählten Referenzgruppe zu bestimmen), dienten jeweils die verfügbaren kumulativen Inzidenzen aller übrigen Kategorien als Referenzkategorie (bspw. Produktionsberufe im Vergleich zu allen anderen Berufen). Damit geben die relativen Risiken darüber Auskunft, wie das durchschnittliche Risiko der Versicherten einer Berufsgruppe im Vergleich zu dem Risiko der jeweils übrigen Berufsgruppen ist [12]. Die Datentabellen wurden von InGef aus der FDB extrahiert und die Berechnungen und Abbildungen erfolgten mit Stata 16.

**Table 2:** 

		Frauen				Männer				
–	–	Anzahl	Fälle	Kum. Inzidenz (pro 100.000)	RR^a^	Anzahl	Fälle	Kum. Inzidenz (pro 100.000)	RR^a^	(KI 95 %)
Berufssektoren	Produktionsberufe	120.429	7699	6393	0,93	779.769	52.585	6744	1,16	(1,15–1,18)
	Personenbezogene Dienstleistungsberufe	470.102	40.569	8630	1,44	141.547	9579	6767	1,09	(1,07–1,11)
	Kaufmännische und unternehmensbez. Dienstleistungsberufe	704.986	41.164	5839	0,75	391.635	21.216	5417	0,83	(0,82–0,85)
	IT- und naturwiss. Dienstleistungsberufe	31.543	1623	5145	0,75	100.682	5161	5126	0,81	(0,79–0,83)
	Sonstige wirtschaftliche Dienstleistungsberufe	113.678	7729	6799	0,99	243.414	14.972	6151	0,98	(0,97–1,00)
Berufssegmente	Land‑, Forst- und Gartenbauberufe	9455	456	4823	0,70	19.051	902	4735	0,76	(0,71–0,81)
	Fertigungsberufe	36.686	2505	6828	1,00	194.998	15.031	7708	1,27	(1,25–1,30)
	Fertigungstechnische Berufe	64.301	4133	6428	0,93	436.764	28.441	6512	1,06	(1,04–1,07)
	Bau- und Ausbauberufe	9987	605	6058	0,88	128.956	8211	6367	1,02	(1,00–1,04)
	Lebensmittel- und Gastgewerbeberufe	68.596	4333	6317	0,92	45.202	2723	6024	0,96	(0,93–1,00)
	Med. u. nicht-med. Gesundheitsberufe	230.748	21.525	9328	1,46	42.062	3397	8076	1,30	(1,26–1,35)
	Soziale und kulturelle Dienstleistungsberufe	170.758	14.711	8615	1,30	54.283	3459	6372	1,02	(0,99–1,05)
	Handelsberufe	182.033	11.493	6314	0,91	112.225	6617	5896	0,94	(0,92–0,96)
	Berufe in Unternehmensführung und -organisation	298.055	16.936	5682	0,79	162.503	8869	5458	0,86	(0,84–0,88)
	Unternehmensbezogene Dienstleistungsberufe	224.898	12.735	5663	0,80	116.907	5730	4901	0,77	(0,75–0,79)
	IT- und naturwiss. Dienstleistungsberufe	31.543	1623	5145	0,75	100.682	5161	5126	0,81	(0,79–0,83)
	Sicherheitsberufe	9264	537	5797	0,84	24.789	1416	5712	0,91	(0,87–0,96)
	Verkehrs- und Logistikberufe	57.226	3686	6441	0,94	206.225	12.810	6212	0,99	(0,98–1,01)
	Reinigungsberufe	47.188	3506	7430	1,09	12.400	746	6016	0,96	(0,90–1,03)
Anforderungsniveau	HelferInnen	199.552	15.364	7699	1,15	181.903	12.599	6926	1,12	(1,10–1,14)
	Fachkräfte	929.056	65.077	7005	1,06	983.958	64.130	6518	1,11	(1,10–1,13)
	SpezialistInnen	173.104	10.283	5940	0,85	269.922	15.581	5772	0,91	(0,90–0,93)
	ExpertInnen	139.066	8061	5797	0,83	221.272	11.204	5063	0,79	(0,77–0,80)
Leitungsfunktion	Aufsichtskräfte	18.517	1107	5978	0,88	60.888	3733	6131	0,99	(0,96–1,02)
	Führungskräfte	21.548	1227	5694	0,83	55.557	2991	5384	0,86	(0,83–0,89)
	Keine Leitungsfunktion	1.448.387	99.061	6839	1,17	1.568.260	97.927	6244	1,08	(1,06–1,11)

*Anzahl* Anzahl der Beobachtungen, *Fälle* Anzahl der Fälle, *kumulative Inzidenzen pro 100.000, RR* relatives Risiko, *KI 95* *%* 95 %-Konfidenzintervalle

^a^Als Referenzgruppe dienen die verfügbaren kumulativen Inzidenzen der übrigen Kategorien

**Table 3:** 

		Frauen					Männer				
–	–	Anzahl	Fälle	Kum. Inzidenz (pro 100.000)	RR^a^	(KI 95 %)	Anzahl	Fälle	Kum. Inzidenz (pro 100.000)	RR^a^	(KI 95 %)
Berufssektoren	Produktionsberufe	120.429	306	254	1,19	(1,06–1,34)	779.769	2790	358	1,14	(1,09–1,21)
	Personenbezogene Dienstleistungsberufe	470.102	1165	248	1,22	(1,14–1,32)	141.547	406	287	0,85	(0,77–0,94)
	Kaufmännische und unternehmensbez. Dienstleistungsberufe	704.986	1179	167	0,63	(0,59–0,68)	391.635	942	241	0,66	(0,62–0,71)
	IT- und naturwiss. Dienstleistungsberufe	31.543	51	162	0,74	(0,56–0,98)	100.682	213	212	0,62	(0,54–0,71)
	Sonstige wirtschaftliche Dienstleistungsberufe	113.678	429	377	1,85	(1,67–2,05)	243.414	1181	485	1,58	(1,48–1,68)
Berufssegmente	Land‑, Forst- und Gartenbauberufe	9455	12	127	0,58	(0,33–1,03)	19.051	47	247	0,74	(0,55–0,98)
	Fertigungsberufe	36.686	91	248	1,15	(0,93–1,41)	194.998	909	466	1,47	(1,37–1,58)
	Fertigungstechnische Berufe	64.301	179	278	1,30	(1,12–1,51)	436.764	1397	320	0,94	(0,89–1,00)
	Bau- und Ausbauberufe	9987	24	240	1,11	(0,74–1,65)	128.956	437	339	1,02	(0,92–1,12)
	Lebensmittel- und Gastgewerbeberufe	68.596	185	270	1,26	(1,08–1,46)	45.202	159	352	1,06	(0,90–1,24)
	Med. u. nicht-med. Gesundheitsberufe	230.748	624	270	1,31	(1,20–1,43)	42.062	120	285	0,85	(0,71–1,02)
	Soziale und kulturelle Dienstleistungsberufe	170.758	356	208	0,95	(0,85–1,07)	54.283	127	234	0,69	(0,58–0,83)
	Handelsberufe	182.033	360	198	0,90	(0,81–1,00)	112.225	288	257	0,76	(0,67–0,85)
	Berufe in Unternehmensführung und -organisation	298.055	475	159	0,69	(0,62–0,76)	162.503	432	266	0,78	(0,71–0,86)
	Unternehmensbezogene Dienstleistungsberufe	224.898	344	153	0,67	(0,60–0,75)	116.907	222	190	0,55	(0,48–0,63)
	IT- und naturwiss. Dienstleistungsberufe	31.543	51	162	0,74	(0,56–0,98)	100.682	213	212	0,62	(0,54–0,71)
	Sicherheitsberufe	9264	27	291	1,34	(0,92–1,96)	24.789	107	432	1,30	(1,07–1,57)
	Verkehrs- und Logistikberufe	57.226	157	274	1,28	(1,09–1,50)	206.225	999	484	1,55	(1,45–1,66)
	Reinigungsberufe	47.188	245	519	2,51	(2,20–2,86)	12.400	75	605	1,82	(1,45–2,29)
Anforderungsniveau	HelferInnen	199.552	746	374	1,95	(1,79–2,11)	181.903	898	494	1,57	(1,46–1,69)
	Fachkräfte	929.056	1888	203	0,84	(0,78–0,90)	983.958	3474	353	1,15	(1,09–1,22)
	SpezialistInnen	173.104	290	168	0,75	(0,66–0,84)	269.922	705	261	0,75	(0,69–0,81)
	ExpertInnen	139.066	206	148	0,66	(0,57–0,76)	221.272	455	206	0,58	(0,53–0,64)
Leitungsfunktion	Aufsichtskräfte	18.517	31	167	0,73	(0,51–1,04)	60.888	173	284	0,83	(0,71–0,96)
	Führungskräfte	21.548	47	218	0,95	(0,71–1,27)	55.557	142	256	0,74	(0,63–0,88)
	Keine Leitungsfunktion	1.448.387	3336	230	1,18	(0,95–1,48)	1.568.260	5441	347	1,28	(1,15–1,44)

*Anzahl* Anzahl der Beobachtungen, *Fälle* Anzahl der Fälle, *kumulative Inzidenzen pro 100.000, RR* relatives Risiko, *KI 95* *%* 95 %-Konfidenzintervalle

^a^Als Referenzgruppe dienen die verfügbaren kumulativen Inzidenzen der übrigen Kategorien

**Table 4:** 

		Frauen					Männer				
–	–	Anzahl	Fälle^a^	Kum. Inzidenz (pro 100.000)	RR^b^	(KI 95 %)	Anzahl	Fälle^a^	Kum. Inzidenz (pro 100.000)	RR^b^	(KI 95 %)
Berufssektoren	Produktionsberufe	120.429	22	18	1,95	(1,24–3,06)	779.769	253	32	1,19	(0,99–1,42)
	Personenbezogene Dienstleistungsberufe	470.102	49	10	1,04	(0,74–1,47)	141.547	23	16	0,52	(0,34–0,80)
	Kaufmännische und unternehmensbez. Dienstleistungsberufe	704.986	47	7	0,49	(0,34–0,69)	391.635	73	19	0,56	(0,44–0,72)
	IT- und naturwiss. Dienstleistungsberufe	31.543	< 5	–	–	–	100.682	16	16	0,52	(0,32–0,85)
	Sonstige wirtschaftliche Dienstleistungsberufe	113.678	25	22	2,41	(1,57–3,72)	243.414	128	53	2,04	(1,67–2,49)
Berufssegmente	Land‑, Forst- und Gartenbauberufe	9455	0	0	–	–	19.051	< 5	–	–	–
	Fertigungsberufe	36.686	9	25	2,51	(1,28–4,93)	194.998	97	50	1,79	(1,43–2,24)
	Fertigungstechnische Berufe	64.301	13	20	2,09	(1,18–3,70)	436.764	116	27	0,83	(0,68–1,02)
	Bau- und Ausbauberufe	9987	0	0	–	–	128.956	37	29	0,94	(0,67–1,31)
	Lebensmittel- und Gastgewerbeberufe	68.596	10	15	1,47	(0,77–2,79)	45.202	11	24	0,79	(0,44–1,44)
	Med. u. nicht-med. Gesundheitsberufe	230.748	22	10	0,93	(0,59–1,46)	42.062	< 5	–	–	–
	Soziale und kulturelle Dienstleistungsberufe	170.758	17	10	0,98	(0,59–1,62)	54.283	8	15	0,48	(0,24–0,96)
	Handelsberufe	182.033	11	6	0,56	(0,30–1,04)	112.225	18	16	0,51	(0,32–0,81)
	Berufe in Unternehmensführung und -organisation	298.055	23	8	0,71	(0,46–1,12)	162.503	35	22	0,68	(0,49–0,97)
	Unternehmensbezogene Dienstleistungsberufe	224.898	13	6	0,53	(0,30–0,93)	116.907	20	17	0,54	(0,35–0,85)
	IT- und naturwiss. Dienstleistungsberufe	31.543	< 5	–	–	–	100.682	16	16	0,51	(0,31–0,83)
	Sicherheitsberufe	9264	0	0	–	–	24.789	15	61	2,02	(1,21–3,37)
	Verkehrs- und Logistikberufe	57.226	12	21	2,16	(1,20–3,91)	206.225	106	51	1,88	(1,52–2,33)
	Reinigungsberufe	47.188	13	28	2,89	(1,63–5,10)	12.400	7	56	1,87	(0,89–3,93)
Anforderungsniveau	HelferInnen	199.552	39	20	2,27	(1,57–3,27)	181.903	83	46	1,64	(1,30–2,08)
	Fachkräfte	929.056	83	9	0,73	(0,52–1,01)	983.958	319	32	1,25	(1,04–1,51)
	SpezialistInnen	173.104	15	9	0,84	(0,49–1,43)	269.922	58	21	0,69	(0,52–0,90)
	ExpertInnen	139.066	9	6	0,61	(0,31–1,21)	221.272	33	15	0,47	(0,33–0,66)
Leitungsfunktion	Aufsichtskräfte	18.517	< 5	–	–	–	60.888	16	26	0,83	(0,50–1,36)
	Führungskräfte	21.548	< 5	–	–	–	55.557	16	29	0,91	(0,55–1,49)
	Keine Leitungsfunktion	1.448.387	168	12	–	–	1.568.260	501	32	1,16	(0,81–1,66)

*Anzahl* Anzahl der Beobachtungen, *Fälle* Anzahl der Fälle, *kumulative Inzidenzen pro 100.000, RR* relatives Risiko, *KI 95* *%* 95 %-Konfidenzintervalle

^a^Werte zwischen 1 und 4 werden in den Daten aus Datenschutzgründen nicht weiter spezifiziert und als „< 5“ ausgegeben. Eine genaue Bestimmung der kumulativen Inzidenz ist daher nicht möglich

^b^Als Referenzgruppe dienen die verfügbaren kumulativen Inzidenzen der übrigen Kategorien

## Ergebnisse

Die Studienpopulation von 3,17 Mio. Versicherten umfasst etwas mehr Männer als Frauen (Tab. 1). Des Weiteren finden sich geschlechterbezogene Unterschiede im Beruf. Männer arbeiten im Vergleich zu Frauen deutlich häufiger in Produktionsberufen sowie in IT- und naturwissenschaftlichen Dienstleistungsberufen, wohingegen Frauen im Vergleich zu Männern häufiger in personenbezogenen, kaufmännischen und unternehmensbezogenen Dienstleistungsberufen arbeiten. Zudem sind Männer vergleichsweise häufiger als Aufsichts- oder Führungskraft tätig und das Anforderungsniveau wird häufiger dem eines Spezialisten oder Experten zugeordnet. Weitere Auswertungen im Onlinematerial (Tabelle S1) zeigen zudem, dass die Verteilung der hier verwendeten Berufsmerkmale gut mit den Daten der Bundesagentur für Arbeit für die Gesamtbevölkerung der Erwerbstätigen in Deutschland übereinstimmt [39].

Die zentralen Ergebnisse zu den beruflichen Unterschieden in Bezug auf COVID-19-Morbidität und -Mortalität sind in den Tab. 2, 3 und 4 dargestellt (jeweils für COVID-19-Erkrankungen, Krankenhausaufenthalte und COVID-19-assoziierte Mortalität). Erhöhte Risiken für COVID-19-Erkrankungen finden sich in personenbezogenen Dienstleistungsberufen oder in medizinischen und nicht-medizinischen Gesundheitsberufen. Das gilt vor allem für Frauen in Gesundheitsberufen, mit relativen Risiken von über 1,4 im Vergleich zu Frauen in übrigen Berufen. Geschlechtsabhängige Unterschiede für ein Erkrankungsrisiko innerhalb von Berufsgruppen finden sich auch bei sozialen und kulturellen Dienstleistungsberufen (höhere Risiken nur für Frauen) und bei Fertigungsberufen (höhere Risiken nur für Männer). Bezüglich des Anforderungsniveaus oder der Leitungsfunktion sind die Ergebnisse für Männer und Frauen ähnlich, mit höchsten Erkrankungsrisiken bei Helfertätigkeiten oder Berufen ohne Leitungsfunktion. Ähnlich sieht es hier in Bezug auf Krankenhausaufenthalt und Mortalität aus. Damit zeichnen sich entlang dieser vertikalen Berufsklassifikationen (insbesondere beim Anforderungsniveau) soziale Gradienten im COVID-19-Geschehen zuungunsten von Erwerbstätigen in niedrig qualifizierten Berufen ab, die bei schweren Krankheitsverläufen besonders deutlich zum Ausdruck kommen. Konkret liegt die kumulative Inzidenz von Krankenhausaufenthalten bei Helfertätigkeiten bei 374 (Frauen) bzw. bei 494 (Männer) und im Vergleich dazu bei Berufen mit Expertentätigkeit bei 148 (Frauen) bzw. bei 206 (Männer). Beim direkten Vergleich dieser 2 Gruppen entspricht dies einem 2,5fach bzw. 2,4fach erhöhtem Risiko für einen Krankenhausaufenthalt (nicht gezeigt in der Tabelle). Allerdings sind die Risiken für einen Krankenhausaufenthalt oder für Mortalität nun vor allem in Reinigungsberufen (insb. bei Frauen) und in Verkehrs- und Logistikberufen (insb. bei Männern) erhöht. Höhere Werte finden sich auch für medizinische und nicht-medizinische Gesundheitsberufe oder für fertigungstechnische Berufe – allerdings nur für Frauen. Trotz der großen Stichprobe ist die Anzahl der Todesfälle insgesamt eher gering. Das gilt vor allem für Frauen und mindert die Aussagekraft der Befunde – obwohl sich die Verteilung der Todesfälle weitestgehend mit der Verteilung der Krankenhausaufenthalte deckt.

**Figure 1:**
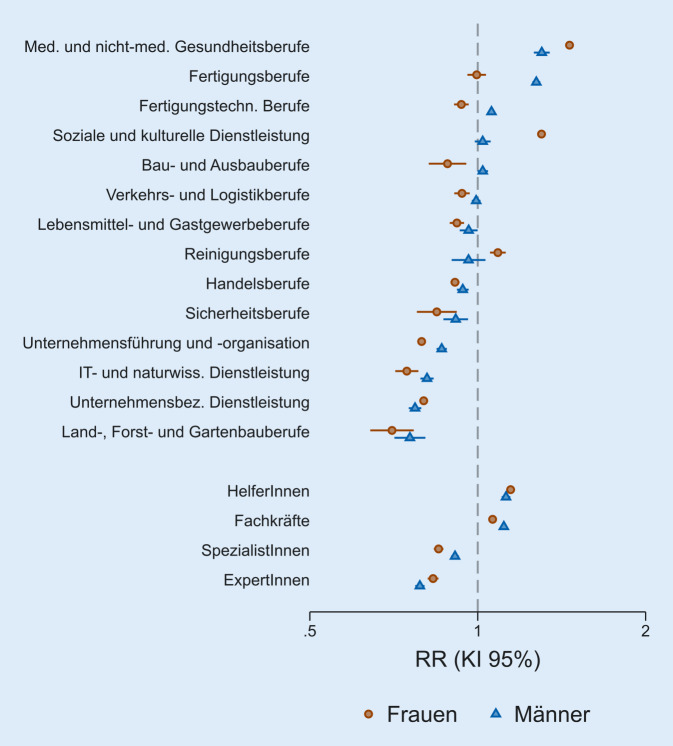

In den Abb. 1 und 2 werden die wichtigsten Ergebnisse anhand der relativen Risiken für Erkrankungsrisiko und Krankenhausaufenthalt zusammengefasst. Dabei sind die Werte für Männer und Frauen direkt gegenübergestellt und in absteigender Reihenfolge sortiert.

**Figure 2:**
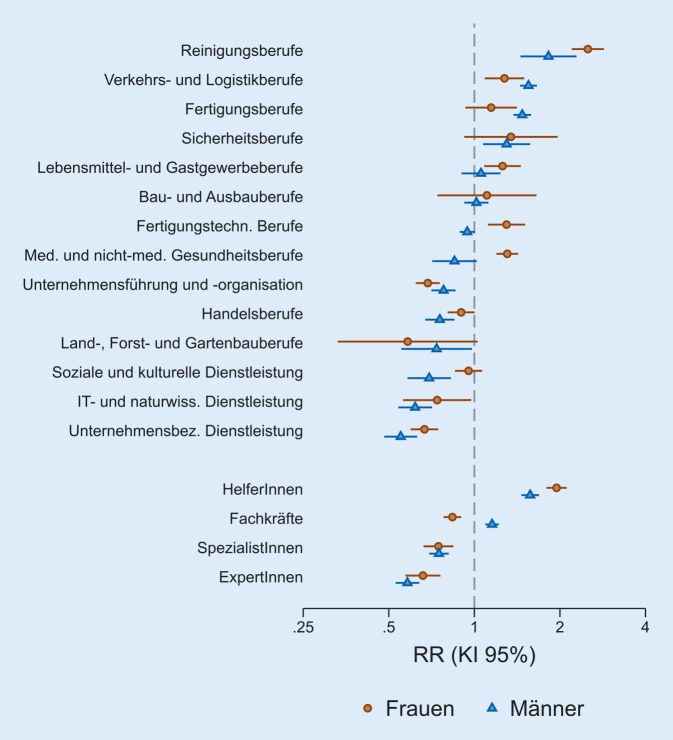

## Diskussion

Im Mittelpunkt der vorliegenden Auswertungen von Krankenkassendaten zu berufsbedingten Unterschieden bei COVID-19-Erkrankungsrisiken, COVID-19-bedingten Krankenhausaufenthalten und COVID-19-assoziierter Mortalität in den ersten 2 Jahren der Pandemie stehen 3 Befunde. Erstens: Der Beruf hat Auswirkung auf das individuelle Risiko für eine COVID-19-Erkrankung. Es sind vor allem personenbezogene Dienstleistungsberufe und Gesundheitsberufe, die ein erhöhtes Erkrankungsrisiko aufweisen. Zudem sind die Risiken für Krankenhausaufenthalt und Mortalität bei Personen in Reinigungsberufen, Verkehrs- und Logistikberufen und in Fertigungsberufen (v. a. für Männer) erhöht. Ein zweiter Befund betrifft Geschlechterunterschiede: In sozialen und kulturellen Dienstleistungsberufen z. B. finden sich erhöhte Erkrankungsrisiken nur bei Frauen und in Fertigungsberufen nur bei Männern. Frauen sind vor allem in Reinigungsberufen von schweren Verläufen betroffen, während es Männer vor allem in Verkehrs- und Logistikberufen sind. Und drittens haben Personen mit Leitungsfunktion und in Berufen mit höherem Anforderungsniveau insgesamt niedrige Risiken für eine Erkrankung oder einen schweren COVID-19-Verlauf. Dabei sind die Risiken bei Expertentätigkeiten am niedrigsten und bei Helfertätigkeiten am höchsten.

Die Befunde stehen damit insgesamt im Einklang mit bisherigen Studien zu sozioökonomischen Unterschieden bei COVID-19-Erkrankungen und Verlaufsschwere [5, 14, 21–24] sowie deutschen ökologischen Studien zu Unterschieden in der regionalen Ausbreitung von Infektionsgeschehen (bzw. Mortalitätsraten) in Abhängigkeit von sozioökonomischen und arbeitsmarktbezogenen Merkmalen von Regionen [14, 25, 26]. Weiter ergänzen die Ergebnisse unserer Studie bisherige Befunde aus Deutschland zu Unterschieden bei COVID-19-bedingter Erwerbsunfähigkeit [27] und liefern erneut Hinweise dafür, dass die Beobachtung eines inversen sozialen Gradienten (d. h. höhere Erkrankungsrisiken für *weniger* benachteiligte Bevölkerungsgruppen oder Regionen) lediglich auf die Anfangsphase der Pandemie beschränkt sein könnte [4, 13]. In der vorliegenden Studie wurde über den Zeitraum von insgesamt 2 Jahren das Fortbestehen eines solchen inversen Zusammenhangs nicht beobachtet. Vielmehr weisen unsere Ergebnisse darauf hin, dass die höheren COVID-19-Risiken in sozioökonomisch benachteiligten Gruppen über den Pandemieverlauf insgesamt überwiegen. Für weitergehende Schlussfolgerungen sind an dieser Stelle allerdings phasenspezifische Analysen notwendig.

Die beobachteten Geschlechterunterschiede deuten zudem an, dass innerhalb der einzelnen Berufsgruppen Unterschiede in den jeweils ausgeübten Tätigkeiten existieren. So kann z. B. bei den Gesundheitsberufen vermutet werden, dass die höheren Risiken bei Frauen auf den vergleichsweise hohen Anteil von Pflegeberufen mit unmittelbarerem Personenkontakt zurückzuführen sind [28], während Männer vergleichsweise häufiger auch Gesundheitsberufe ohne Personenkontakt haben bzw. eine Position mit geringerem Erkrankungsrisiko einnehmen (höherer Anteil von Männern in leitenden Gesundheitsberufen). Damit wären Frauen in Gesundheitsberufen nicht nur häufiger gegenüber dem Virus exponiert, sondern im Fall einer Erkrankung auch vulnerabler für schwere Verläufe [2]. Ähnliches gilt vermutlich auch für die Gruppe der Männer in Verkehrs- und Logistikberufen bzw. in Fertigungsberufen. Neben der fehlenden Möglichkeiten zur Arbeit im Homeoffice kann hier vermutet werden, dass die Zahl der Mitarbeitenden in unmittelbarer Umgebung größer und die Umsetzung von Infektionsschutzmaßnahmen (z. B. das Einhalten von Abstandsregeln) schwieriger ist [29]. Denkbar ist auch, dass Berufsgruppen mit höheren Infektions- bzw. Erkrankungsrisiken insgesamt auch seltener von Schließungen im Rahmen der Infektionsschutzmaßnahmen betroffen waren und dadurch Expositionen gegenüber dem Virus *per se* (bei der Arbeit, aber auch bei der Nutzung des öffentlichen Nahverkehrs auf dem Weg zur Arbeit) höher waren. Eine abschließende Antwort auf diese Fragen war in der vorliegenden Studie nicht möglich und erfordert auch die Berücksichtigung von Merkmalen (z. B. Details zu Arbeitsbedingungen), die nicht Teil der Routinedaten von Krankenkassen sind [30].

### Einschränkungen

Trotz hoher Fallzahl und der Analyse von Individualdaten getrennt nach Männern und Frauen sowie ärztlicher Diagnosen zu den Outcomes (etwa im Gegensatz zu selbstberichteten Infektionen in Befragungen) bleiben die Möglichkeiten einer vertieften Analyse zu den Ursachen der beschriebenen Zusammenhänge auf Basis von Routinedaten beschränkt. In diesem Zusammenhang gilt auch, dass mögliche Confounder mit den vorliegenden Routinedaten nur unzureichend berücksichtigt werden können. Zum Beispiel könnte ein Migrationsstatus von Versicherten sowohl für COVID-19-Risiken eine Rolle spielen [31–33] als auch in enger Beziehung zum Beruf stehen. Diese Information ist allerdings kein Teil der verwendeten Krankenkassendaten. Ebenfalls könnte in unseren Analysen das Alter ein Confounder sein – z. B. wenn tendenziell ältere Menschen in Fertigungsberufen arbeiten und gleichzeitig ein höheres Risiko für einen schweren COVID-19-Verlauf haben. Weiterführende Sensitivitätsanalysen auf Basis altersstandardisierter kumulativer Inzidenzen (mittels direkter Standardisierung auf Basis der überarbeiteten europäischen Standardbevölkerung [34]) decken sich allerdings mit unseren Befunden (siehe Onlinematerial Tabelle S2 für Details).

Besondere Vorsicht ist auch bei der Interpretation der Ergebnisse im Falle von Krankenhausaufenthalten und COVID-19-assoziierter Mortalität geboten [35]. Sowohl bei den Krankenhausaufenthalten als auch bei der COVID-19-assoziierten Mortalität ist eine Erkrankung quasi Voraussetzung für einen schweren Verlauf, weshalb es sich bei den untersuchten Merkmalen um eine Kombination des Risikos einer Infektion bzw. Erkrankung und des Risikos eines schweren Verlaufes nach einer Infektion handelt. Daher präsentieren wir im Onlinematerial zusätzlich noch Zusammenhangsanalysen auf Basis der Versicherten mit einer COVID-19-Diagnose (Tabellen S3 und S4 im Onlinematerial), die zusätzlich auch Aussagen zum Risiko derjenigen mit einer Infektion ermöglichen (also die „case fatality rate“ im Falle von Mortalität). Die Ergebnisse bestätigen, dass Krankenhausaufenthalte oder Mortalität nicht nur insgesamt, sondern insbesondere auch im Falle einer Infektion für die betroffenen Berufsgruppen erhöht sind. Einschränkend muss die Messung der 3 COVID-19-bezogenen Outcomes reflektiert werden. Denn bei den COVID-19-Erkrankungen ist – obwohl sie auf laborbestätigten, an die Krankenkasse übermittelten Erkrankungen beruhen – nicht auszuschließen, dass bestimmte Berufsgruppen auch aufgrund von Unterschieden im Gesundheitsverhalten (z. B. verspätete Symptomwahrnehmung und spätere Inanspruchnahme von Hilfe) seltener eine COVID-19-Erkrankung aufweisen [36, 37]. Dies könnte dazu führen, dass bestimmte Berufsgruppen trotz eines positiven Selbsttests häufiger keinen Labortest durchführen lassen. So schätzte eine deutsche Studie auf der Basis eines Vergleichs von nachgewiesenen Antikörpern im Blut und regionalen Meldedaten zu Infektionen (vor der Verfügbarkeit von Impfstoffen; [38]), dass bis zu 45 % der SARS-CoV-2-Infektionen unentdeckt blieben, mit etwas höheren Werten in sozioökonomisch benachteiligten Regionen. Vor diesem Hintergrund ist es durchaus möglich, dass das Erkrankungsrisiko für bestimmte Berufsgruppen (z. B. Reinigungsberufe, Verkehrs- und Logistikberufe, aber auch für Land‑, Forst- und Gartenbauberufe) in unserer Studie eher unterschätzt wird. Auch bei den betrachteten COVID-19-assoziierten Krankenhausaufenthalten ist es denkbar, dass bestimmte Berufsgruppen eine ambulante Behandlung bevorzugten und somit COVID-19-Diagnosen mit einem gewissen Schweregrad und Behandlungsbedarf (aber ohne Krankenhausaufenthalt) in unserer Studie nicht berücksichtigt wurden. Darüber hinaus wurde bei der Ermittlung der COVID-19-Outcomes nicht zwischen Haupt- und Nebendiagnosen unterschieden. COVID-19-Krankenhausaufenthalte und COVID-19-assoziierte Mortalität sind daher, wenn sie über eine Nebendiagnose ermittelt werden, nur eingeschränkt ein Indikator für die Verlaufsschwere, z. B. wenn ein Patient mit einem Herzinfarkt ins Krankenhaus eingeliefert wurde und bei der Aufnahme COVID-19 diagnostiziert wurde. Es ist jedoch unklar, warum ein solcher Fall (Bestimmung über Nebendiagnosen) zwischen den Berufsgruppen variieren sollte und somit für die in der vorliegenden Studie gefundenen Unterschiede relevant ist. Schließlich muss festgehalten werden, dass es sich in der vorliegenden Studie um eine deskriptive Studie mit einer systematischen Beschreibung von COVID-19-bezogenen Risiken entlang von Berufsmerkmalen handelt, nicht aber um eine weitergehende Analyse zugrunde liegender Mechanismen auf Basis weiterführender statistischer Verfahren (bspw. multivariable Überlebendzeitmodelle unter Kontrolle verfügbarer möglicher Störgrößen sowie gleichzeitige Betrachtung der Berufsmerkmale).

## Fazit

Die vorliegende Studie liefert einen wichtigen Beitrag zum aktuellen Forschungsstand zu berufsbedingten Unterschieden in COVID-19-Morbidität und -Mortalität in Deutschland. Der Beitrag unterstreicht dabei insgesamt die Bedeutung des Berufs und deutet auch erstmalig an, dass Zusammenhänge nach Geschlecht variieren können. Damit liefert der Beitrag wichtige Anhaltspunkte dafür, dass die Arbeitswelt ein wichtiger Ansatzpunkt zur Entwicklung von Infektionsschutzmaßnahmen ist und deren Berücksichtigung auch stärkerer Bestandteil künftiger Pandemiepläne sein sollte. Für das Management zukünftiger Pandemien bedeutet dies beispielsweise, dass – wo es möglich ist – rechtliche Rahmenbedingungen und technische Vorrausetzungen für die Möglichkeiten von Homeoffice als Vorsorge verbessert werden sollten. Weiter deuten unsere Befunde an, dass eine Verringerung existierender sozialer Gesundheitsungleichheiten auch eine Verringerung beobachteter beruflicher Ungleichheiten in der Verlaufsschwere bedeutet.

## Supplementary Information




