# Supplementary material for: Occupational differences of COVID-19 morbidity and mortality in Germany. An analysis of health insurance data from 3.17 million insured persons
Source: Bundesgesundheitsblatt Gesundheitsforschung Gesundheitsschutz. 2023 Jul 19;66(8):857–68. [Article in German] doi: 10.1007/s00103-023-03738-9 (PMC10371894; doi:10.1007/s00103-023-03738-9)
Supplement: Supplementary file 1 [file 103_2023_3738_MOESM1_ESM.pdf]

Berufsbedingte Unterschiede bei COVID-19-Morbidität und -Mortalität in Deutschland.

Eine Analyse von Krankenkassendaten von 3,17 Mio. Versicherten

Morten Wahrendorf[1], Valerie Schaps[1], Marvin Reuter[2], Jens Hoebel[3], Benjamin Wachtler[3], Josephine Jacob[4], Marco Alibone[4], Nico Dragano[1]

[1] Institut für Medizinische Soziologie, Centre for Health and Society, Medizinische Fakultät und Universitätsklinikum, Universität Düsseldorf, Deutschland

[2] Juniorprofessur für Soziologie, insb. Arbeit und Gesundheit, Fakultät für Wirtschafts- und Sozialwissenschaften, Otto-Friedrich-Universität Bamberg, Deutschland

[3] Fachgebiet Soziale Determinanten der Gesundheit, Abteilung für Epidemiologie und Gesundheitsmonitoring, Robert Koch-Institut, Berlin, Deutschland

[4] InGef - Institut für angewandte Gesundheitsforschung, Berlin GmbH, Berlin, Deutschland

Onlinematerial

Abbildung S1: Flussdiagramm zur Auswahl der Studienpopulation. Quelle: Eigene Abbildung.

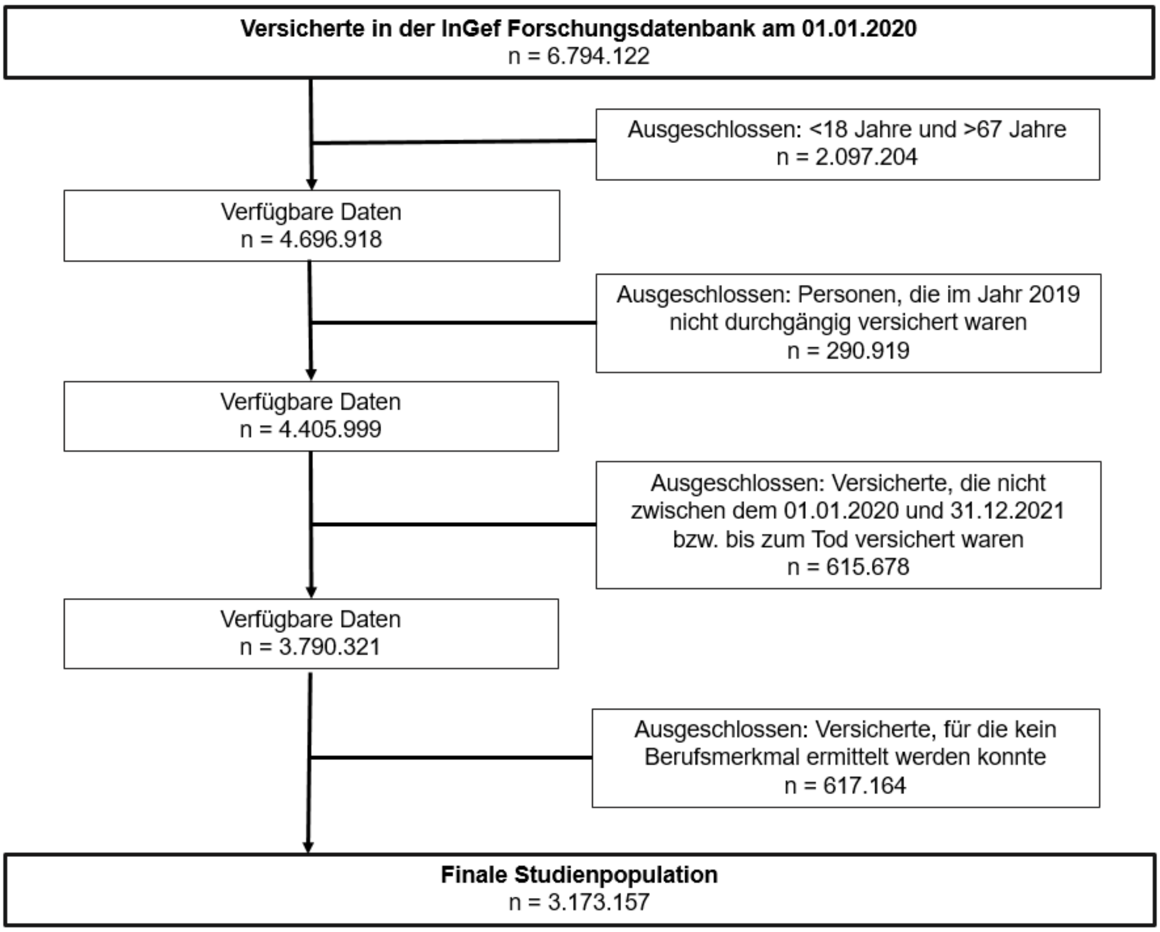

Tabelle S1. Vergleich Sozialversicherungspflichtig Beschäftigte und Studienpopulation. Werte für Gesamtbevölkerung beruhen auf eigenen Berechnung auf Basis der Angaben für Dezember 2019 der Bundesagentur für Arbeit für Sozialversicherungspflichtig Beschäftige [39].

	Kategorie oder Rangweite	Studien-population (%)	Gesamt-bevölkerung (%)
Berufssektoren	Produktionsberufe	29,1	26,7
	Personenbezogene Dienstleistungsberufe	19,7	24,1
	Kaufmännische und unternehmensbezogene Dienstleistungsberufe	35,4	31,7
	IT- und naturwissenschaftliche Dienstleistungsberufe	4,3	4,0
	Sonstige wirtschaftliche Dienstleistungsberufe	11,5	13,5
Berufssegmente	Land-, Forst- und Gartenbauberufe	0,9	1,4
	Fertigungsberufe	7,5	7,1
	Fertigungstechnische Berufe	16,2	12,2
	Bau- und Ausbauberufe	4,5	6,0
	Lebensmittel- und Gastgewerbeberufe	3,7	5,0
	Medizinische u. nicht-medizinische Gesundheitsberufe	8,8	10,8
	Soziale und kulturelle Dienstleistungsberufe	7,3	8,3
	Handelsberufe	9,5	9,5
	Berufe in Unternehmensführung und -organisation	14,9	12,9
	Unternehmensbezogene Dienstleistungsberufe	11,0	9,2
	IT- und naturwissenschaftliche Dienstleistungsberufe	4,3	4,0
	Sicherheitsberufe	1,1	1,1
	Verkehrs- und Logistikberufe	8,5	9,8
	Reinigungsberufe	1,9	2,6
Anforderungsniveau	HelferInnen	12,3	15.3
	Fachkräfte	61,8	58.3
	SpezialistInnen	14,3	12,9
	ExpertInnen	11,6	13,5
Leitungsfunktion	Aufsichts- oder Führungskräfte	4,9	5,1
	Keine Leitungsfunktion	95,1	94,9

Tabelle S2. Altersstandardisierte kumulative Inzidenzen (ASKI) nach Berufsmerkmalen für die drei Outcomes COVID-19-Erkrankung, Krankenhausaufenthalt und Mortalität. Die Altersstandardisierung erfolgt auf Basis der jeweiligen Inzidenzen in fünf Altersgruppen („18 – 29“, „30 – 39“, „40 – 49“, „50 – 59“, und „60 – 67“). Aufgrund geringer Fallzahlen für Krankenhausaufenthalt und Mortalität, bei denen Werte zwischen 1 und 4 aus Datenschutzgründen nicht weiter spezifiziert werden, können in diesen Fällen keine Altersstandardisierte kumulative Inzidenzen bestimmt werden.

		Frauen			Männer		
		Erkrankung	KH-Aufenthalt	Mortalität	Erkrankung	KH-Aufenthalt	Mortalität
		ASKI	ASKI	ASKI	ASKI	ASKI	ASKI
Berufssektoren	Produktionsberufe	6.365	253	.	6.819	334	.
	Personenbezogene Dienstleistungsberufe	8.507	253	11	6.680	319	.
	Kaufmännische und unternehmensbez. Dienstleistungsberufe	5.941	167	.	5.466	233	.
	IT- und naturwiss. Dienstleistungsberufe	5.090	169	.	5.079	220	.
	Sonstige wirtschaftliche Dienstleistungsberufe	7.075	352	.	6.358	429	.
Berufssegmente	Land-, Forst- und Gartenbauberufe	4.746	.	0	4.697	.	.
	Fertigungsberufe	6.838	238	.	7.828	425	.
	Fertigungstechnische Berufe	6.421	277	.	6.591	302	.
	Bau- und Ausbauberufe	5.882	.	0	6.422	313	.
	Lebensmittel- und Gastgewerbeberufe	6.285	271	15	5.937	402	.
	Med. u. nicht-med. Gesundheitsberufe	9.154	281	.	7.913	317	.
	Soziale und kulturelle Dienstleistungsberufe	8.533	210	.	6.336	253	.
	Handelsberufe	6.375	198	.	5.854	257	.
	Berufe in Unternehmensführung und -organisation	5.813	154	.	5.546	249	.
	Unternehmensbezogene Dienstleistungsberufe	5.742	156	.	4.968	182	.
	IT- und naturwiss. Dienstleistungsberufe	5.090	169	.	5.079	220	.
	Sicherheitsberufe	6.071	.	0	6.229	.	.
	Verkehrs- und Logistikberufe	6.597	266	.	6.403	429	.
	Reinigungsberufe	7.970	507	.	6.086	572	.
Anforderungsniveau	HelferInnen	7.873	353	.	6.917	487	.
	Fachkräfte	7.025	204	.	6.578	339	.
	SpezialistInnen	5.889	174	.	5.831	239	.
	ExpertInnen	5.710	154	.	5.045	196	.
Leitungsfunktion	Aufsichtskräfte	5.922	.	.	6.308	.	.
	Führungskräfte	5.922	.	.	6.019	.	.
	Keine Leitungsfunktion	6.881	229	.	6.283	333	31

Tabelle S3. COVID-19-Krankenhausaufenthalt nach Berufsmerkmalen für Frauen und Männer mit SARS-CoV-2-Infektion: Anzahl der Beobachtungen (Anzahl), Anzahl der Fälle (Fälle), kumulative Inzidenzen pro 100.000.

		Frauen			Männer		
		Anzahl	Fälle	Kum. Inzidenz (pro 100.000)	Anzahl	Fälle	Kum. Inzidenz (pro 100.000)
Berufssektoren	Produktionsberufe	7.699	306	3.975	52.585	2790	5.306
	Personenbezogene Dienstleistungsberufe	40.569	1165	2.872	9.579	406	4.238
	Kaufmännische und unternehmensbez. Dienstleistungsberufe	41.164	1179	2.864	21.216	942	4.440
	IT- und naturwiss. Dienstleistungsberufe	1.623	51	3.142	5.161	213	4.127
	Sonstige wirtschaftliche Dienstleistungsberufe	7.729	429	5.551	14.972	1181	7.888
Berufssegmente	Land-, Forst- und Gartenbauberufe	456	12	2.632	902	47	5.211
	Fertigungsberufe	2.505	91	3.633	15.031	909	6.048
	Fertigungstechnische Berufe	4.133	179	4.331	28.441	1397	4.912
	Bau- und Ausbauberufe	605	24	3.967	8.211	437	5.322
	Lebensmittel- und Gastgewerbeberufe	4.333	185	4.270	2.723	159	5.839
	Med. u. nicht-med. Gesundheitsberufe	21.525	624	2.899	3.397	120	3.533
	Soziale und kulturelle Dienstleistungsberufe	14.711	356	2.420	3.459	127	3.672
	Handelsberufe	11.493	360	3.132	6.617	288	4.352
	Berufe in Unternehmensführung und -organisation	16.936	475	2.805	8.869	432	4.871
	Unternehmensbezogene Dienstleistungsberufe	12.735	344	2.701	5.730	222	3.874
	IT- und naturwiss. Dienstleistungsberufe	1.623	51	3.142	5.161	213	4.127
	Sicherheitsberufe	537	27	5.028	1.416	107	7.556
	Verkehrs- und Logistikberufe	3.686	157	4.259	12.810	999	7.799
	Reinigungsberufe	3.506	245	6.988	746	75	10.054
Anforderungsniveau	HelferInnen	15.364	746	4.856	12.599	898	7.128
	Fachkräfte	65.077	1888	2.901	64.130	3474	5.417
	SpezialistInnen	10.283	290	2.820	15.581	705	4.525
	ExpertInnen	8.061	206	2.556	11.204	455	4.061
Leitungsfunktion	Aufsichtskräfte	1.107	31	2.800	3.733	173	4.634
	Führungskräfte	1.227	47	3.830	2.991	142	4.748
	Keine Leitungsfunktion	99.061	3336	3.368	97.927	5441	5.556

Tabelle S4. COVID-19-Mortalität nach Berufsmerkmalen für Frauen und Männer mit SARS-CoV-2-Infektion: Anzahl der Beobachtungen (Anzahl), Anzahl der Fälle (Fälle), kumulative Inzidenzen pro 100.000.

		Frauen			Männer		
		Anzahl	Fälle	Kum. Inzidenz (pro 100.000)	Anzahl	Fälle	Kum. Inzidenz (pro 100.000)
Berufssektoren	Produktionsberufe	7.699	22	286	52.585	253	481
	Personenbezogene Dienstleistungsberufe	40.569	49	121	9.579	23	240
	Kaufmännische und unternehmensbez. Dienstleistungsberufe	41.164	47	114	21.216	73	344
	IT- und naturwiss. Dienstleistungsberufe	1.623	<5	.	5.161	16	310
	Sonstige wirtschaftliche Dienstleistungsberufe	7.729	25	323	14.972	128	855
Berufssegmente	Land-, Forst- und Gartenbauberufe	456	0	0	902	<5	.
	Fertigungsberufe	2.505	9	359	15.031	97	645
	Fertigungstechnische Berufe	4.133	13	315	28.441	116	408
	Bau- und Ausbauberufe	605	0	0	8.211	37	451
	Lebensmittel- und Gastgewerbeberufe	4.333	10	231	2.723	11	404
	Med. u. nicht-med. Gesundheitsberufe	21.525	22	102	3.397	<5	.
	Soziale und kulturelle Dienstleistungsberufe	14.711	17	116	3.459	8	231
	Handelsberufe	11.493	11	96	6.617	18	272
	Berufe in Unternehmensführung und -organisation	16.936	23	136	8.869	35	395
	Unternehmensbezogene Dienstleistungsberufe	12.735	13	102	5.730	20	349
	IT- und naturwiss. Dienstleistungsberufe	1.623	<5	.	5.161	16	310
	Sicherheitsberufe	537	0	0	1.416	15	1.059
	Verkehrs- und Logistikberufe	3.686	12	326	12.810	106	827
	Reinigungsberufe	3.506	13	371	746	7	938
Anforderungsniveau	HelferInnen	15.364	39	254	12.599	83	659
	Fachkräfte	65.077	83	128	64.130	319	497
	SpezialistInnen	10.283	15	146	15.581	58	372
	ExpertInnen	8.061	9	112	11.204	33	295
Leitungsfunktion	Aufsichtskräfte	1.107	<5	.	3.733	16	429
	Führungskräfte	1.227	<5	.	2.991	16	535
	Keine Leitungsfunktion	99.061	168	170	97.927	501	512
